# A case of esophageal squamous cell carcinoma with epidermization showing a unique morphology

**DOI:** 10.1007/s12328-024-02042-6

**Published:** 2024-10-18

**Authors:** Jyunichi Mizuno, Yuji Urabe, Hikaru Nakahara, Ken Yamashita, Yuichi Hiyama, Hidehiko Takigawa, Akira Ishikawa, Toshio Kuwai, Takao Hinoi, Shiro Oka

**Affiliations:** 1 Department of Gastroenterology, Hiroshima University Hospital, 1-2-3, Kasumi, Minamiku, Hiroshima, 734-8551 Japan; 2 Department of Gastroenterology, Graduate School of Biomedical and Health Sciences, Hiroshima University, Hiroshima, Japan; 3 Department of Clinical and Molecular Genetics, Hiroshima University Hospital, Hiroshima, Japan; 4 Department of Molecular Pathology, Graduate School of Biomedical and Health Sciences, Hiroshima University, Hiroshima, Japan; 5 Department of Gastrointestinal Endoscopy and Medicine, Hiroshima University Hospital, Hiroshima, Japan

**Keywords:** Epidermoid metaplasia of esophagus, Esophageal squamous cell carcinoma, Cancer genomic testing

## Abstract

An 80-year-old woman with a history of endoscopic balloon dilation for esophageal stricture caused by accidental ingestion of caustic soda during infancy presented with dysphagia. Upper gastrointestinal endoscopy revealed a 10-cm-long, highly white, elevated lesion with a feathered appearance. This lesion was determined to be the cause of dysphagia and was completely resected via endoscopic submucosal dissection. Histopathological examination revealed a thick keratin layer on the surface of the stratified squamous epithelium, with a prominent granular layer underneath and some areas showing nuclear atypia. The lesion was diagnosed as a well-differentiated squamous cell carcinoma, pT1a-LPM, derived from epidermoid metaplasia. Cancer genome analysis revealed mutations in *TP53* as well as amplification of *MYC*, *FGFR1*, chromosome 7, and chromosome 20q. This case suggests that epidermoid metaplasia caused by chronic irritation from an esophageal stricture may have been exacerbated by the dilation procedure.

**Supplementary Information:**

The online version contains supplementary material available at 10.1007/s12328-024-02042-6.

## Introduction

Epidermoid metaplasia (EM) is a rare pathological condition in which the normal, non-keratinized squamous epithelium of the esophagus transforms into a keratinized, skin-like epithelium. This condition can be caused by chronic irritation or injury to the esophageal lining, such as that caused by chronic gastroesophageal reflux disease, caustic injury, chronic inflammation, or vitamin deficiencies [1, 2]. EM involves pathological changes in the esophageal mucosa, characterized histologically by a thick keratin layer on the surface of the squamous epithelium, resembling the epidermis of the skin. In endoscopic findings of esophageal EM (EEM), the keratinized epithelium appears as white, scaly, or shaggy keratin layers that seem to be attached to the esophageal mucosa [3]. In addition, EEM has been reported as a potential premalignant condition for esophageal cancer [4].

Here, we report a case of superficial esophageal squamous cell carcinoma (ESCC) with EM showing a unique morphology.

## Case report

The present case involves an 80-year-old female who was clinically diagnosed with ESCC. She had a history of accidental ingestion of caustic soda as a child (at 1 year of age). The patient has no history of alcohol consumption or smoking. In August 2012, the patient visited a clinic with the chief complaint of dysphagia. Esophagogastroduodenoscopy (EGD) revealed esophageal stenosis 20 cm from the incisors, surrounded by a white flattened, elevated lesion. There were no findings suggestive of reflux esophagitis. Consequently, the patient was referred to our hospital for thorough examination and treatment of the esophageal stricture.

**Fig. 1:**
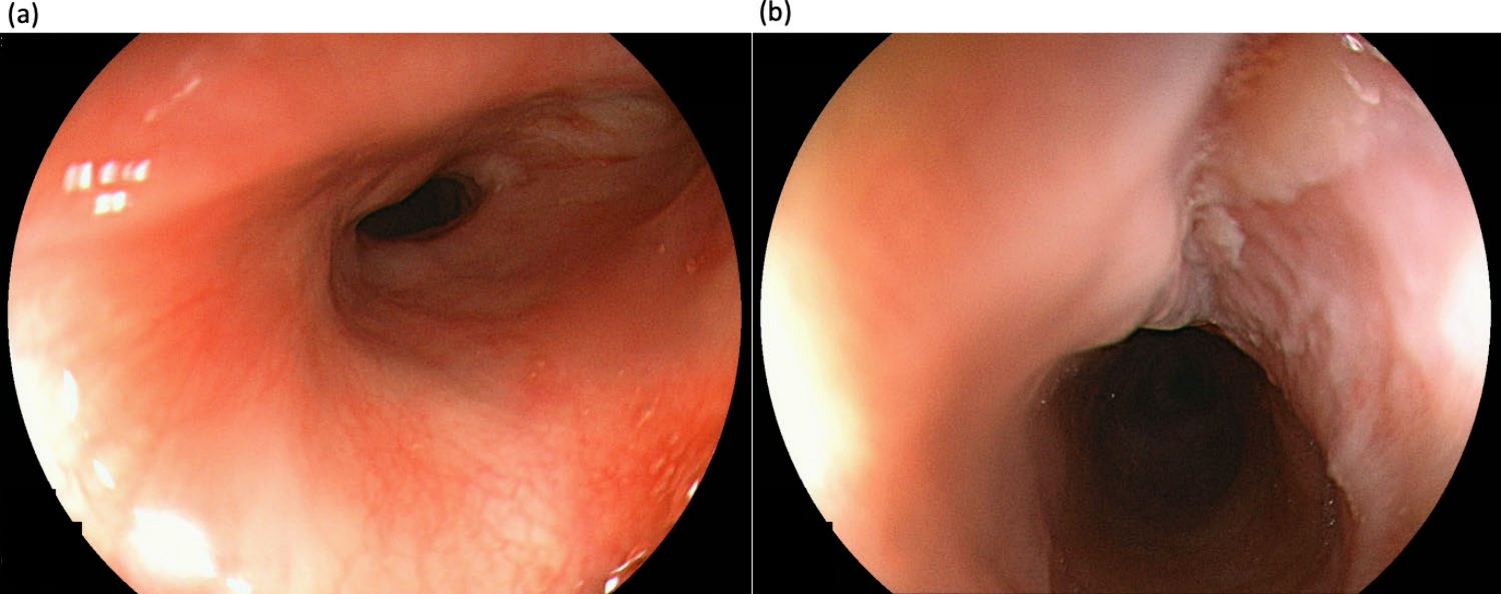
The endoscopic image from 8 years ago of the site where the tumor was localized. The endoscopic image from 8 years ago did not reveal any tall elevated lesion. **a** Panoramic view of the stenotic area. **b** A close-up image of the stenotic area reveals a whitish, flat elevation on the oral side of the stenosis

During EGD in our department, the stenosis prevented the passage of a normal-sized oral endoscope with a diameter of approximately 1 cm (GIF-260 J, Olympus, Tokyo, Japan) (Fig. 1). Thirteen endoscopic balloon dilations (EBDs) of the stenoses were performed in April 2013. Once the dysphagia had improved, dilation was considered completed, and the patient was monitored. In November 2013, tongue cancer was diagnosed, and pathology results showed epithelial dysplasia carcinoma in situ, which was treated with small-source therapy using Au grains.

**Fig. 2:**
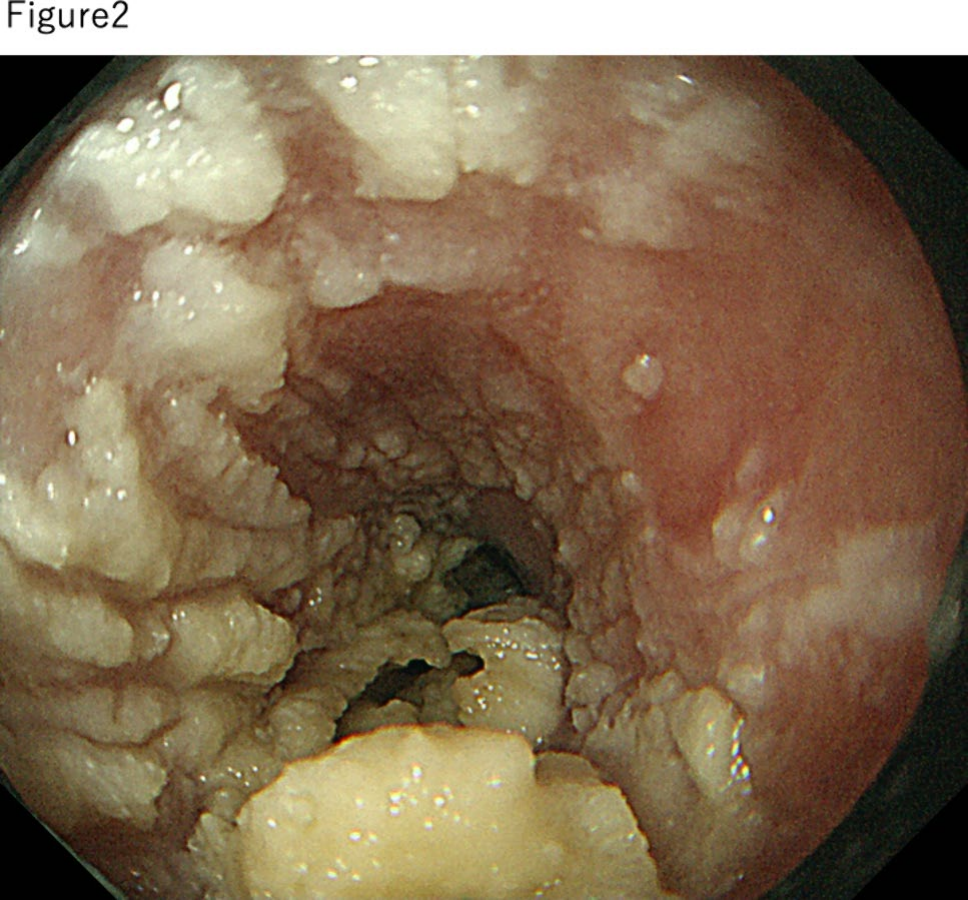
Endoscopic findings of esophageal stenosis observed 2 years before endoscopic treatment when the patient presented again with dysphagia

In July 2018, the patient presented with dysphagia again, and EGD was repeated. This revealed a three-quarters circumferential white-toned, elevated lesion at 25–30 cm from the upper incisor dentition, with a flat elevation on the oral side, a taller elevation on the anal side, and narrowing of the lumen in the region (Fig. 2). The whitish ridges did not stain with iodine. Malignancy was considered a differential diagnosis; however, histopathological examination of the biopsy specimen showed that the basal layer of the superficial epithelium exhibited regenerative changes, and the superficial layer was hyperkeratotic with no evidence of malignancy. Three EBDs were performed, which did not alleviate the esophageal stenosis, and it was decided to monitor the patient after consultation.

**Fig. 3:**
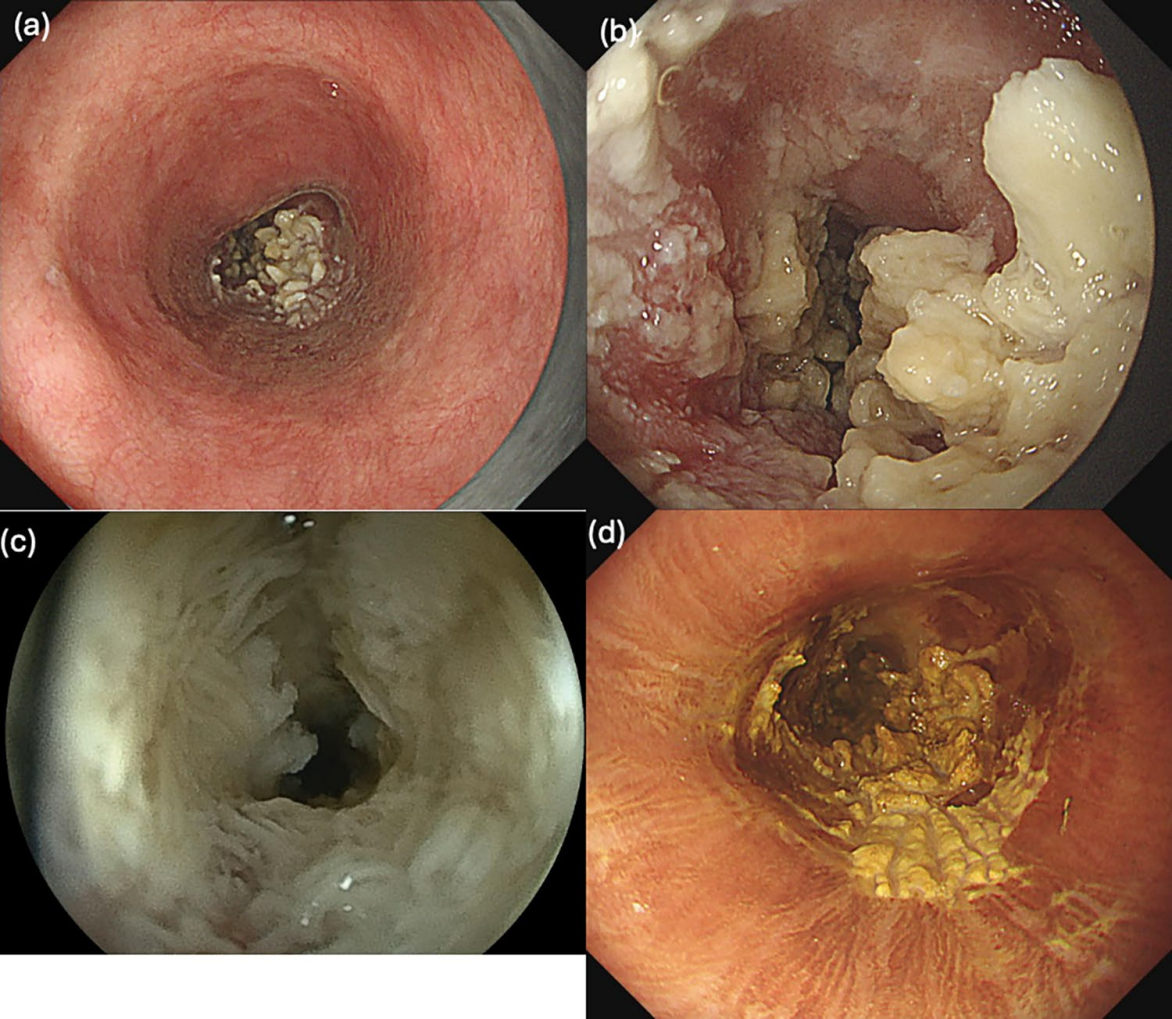
Endoscopic findings of the lesion at endoscopic submucosal dissection. **a** Panoramic view of the lesion. **b** A close-up image of the lesion. In the lesion, a whitish, high-evaluated area and a squamous, flat elevated area are observed. **c** The high-evaluated area was stenotic. With a thin-diameter endoscope, a fringed keratinized substance is observed on the elevated area. **d** This lesion exhibits mild to lack of staining with Lugol’s iodine

**Fig. 4:**
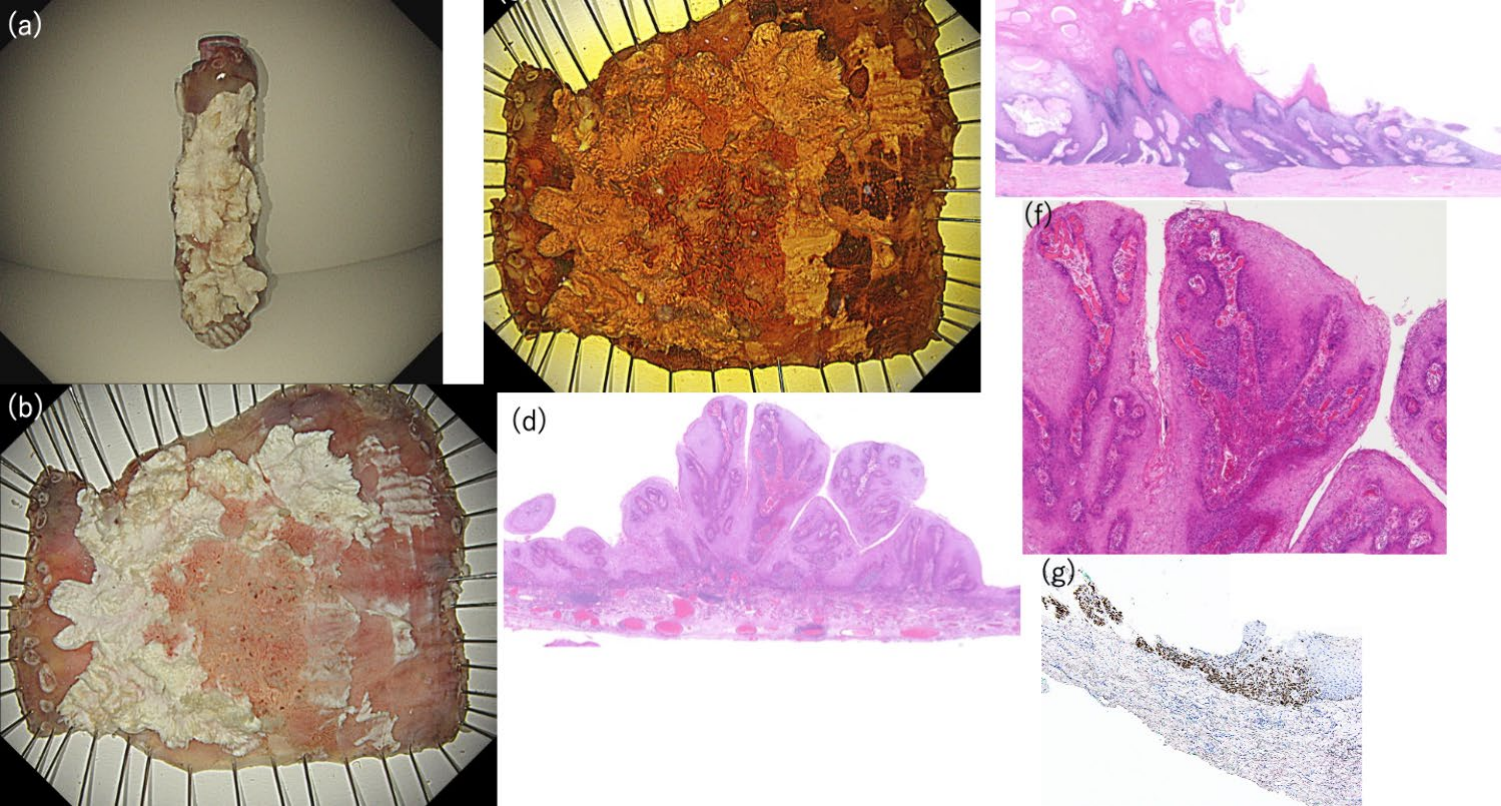
Macroscopic and pathological findings of endoscopic submucosal dissection (ESD) specimens. **a** The tumor was resected in a tubular fashion using ESD. **b** Gross finding after formalin fixation following ESD. **c** Iodine-stained findings of the resected specimen by ESD. **d** Histopathological findings of the elevated area (low magnification view). **e** At the margin of the elevated area, a distinctly separated thickened keratin layer and granular layer are observed, and these layers are clearly demarcated (low magnification view). **f** Exophytic and papillary proliferation of low-grade tumor cells within the stratified squamous epithelium is observed. **g** Immunohistochemical findings for p53: p53-positive staining is observed in a subset of tumor cells

In June 2023, during a screening EGD, biopsy tissue from this lesion showed atypical epithelium, and the patient was referred to us again. In the same month, EGD showed a complete circumferential white keratinizing epithelium with thick furrowing at 21–30 cm, a flat elevated white adherence at 25 cm from the incisors, and narrowing from 28 to 30 cm from the incisors, which did not allow passage of a normal-diameter scope (Fig. 3). The scope was changed to a narrow-diameter scope to observe the anorectal side. Histopathology showed that the epithelium was extraordinarily papillary, with hyperkeratosis and calcified degeneration in the superficial layers. The biopsy results showed no evidence of malignancy; however, the lesion was considered the cause of dysphagia; therefore, it was resected en bloc by endoscopic submucosal dissection (ESD) (Fig. 4a–c). After ESD, to prevent esophageal restenosis, 400 mg of triamcinolone was injected into the ulcer base three times: immediately after treatment, on the day after treatment, and 7 days after treatment. In addition, oral prednisolone was administered at 30 mg/day for 1 week, followed by dose reduction by 5 mg each week for 6 weeks. Despite this, dysphagia with a Dysphagia Score of 2 developed in November 2023, and EGD confirmed stenosis. Consequently, to relieve the stenosis, a total of 21 EBD procedures were performed starting in November 2023. Finally, the stenosis was resolved by June 2024.

The pathological findings of the ESD specimen showed a circumferential, white-toned, superficial elevated tumor, with a few atypical tumor cells showing exotropia and papillary growth within the stratified squamous epithelium and a thickened stratum corneum and granular layer on the tumor surface, which were distinctly separated from each other. Some of these layers were delaminating (Fig. 4d–f). Immunohistochemistry of this lesion showed positive results for p53, localized to the tumor area of the moderately differentiated component (Fig. 4g). The pathological diagnosis of this lesion was squamous cell carcinoma (pT1a-LPM, Ly0, v0, HM0, VM0) and ESCC with EEM.

We performed cancer multigene panel testing to investigate the etiology of the lesions. Formalin-fixed, paraffin-embedded (FFPE) specimens of the high-elevated area of the ESD specimen of this lesion were dissected into 10 µm-thick tumor tissue sections and mounted on microscopic slides, and DNA was extracted using a QIAamp DNA FFPE Tissue kit (QIAGEN, Valencia, CA, USA) according to the manufacturer’s instructions. DNA from this lesions and blood cells was confirmed to be of sufficient quality and quantity for next-generation sequence library calculations using Qubit 1.0 Fluorometer (Life Technologies, Grand Island, NY, USA) and Genomic DNA ScreenTape Analysis (Agilent Technologies, Santa Clara, CA, USA). Next, sequence libraries mounted on 468 cancer-related genes from the MSK-IMPACT Clinical Sequencing Cohort (Supplementary Table 1) were prepared using DNA from the lesions and blood cells using our sequence library method. The resulting pooled libraries were sequenced using paired-end reads on the HiSeq X platform (Illumina, San Diego, CA, USA). Sequencing reads were analyzed and annotated as described in the Supplementary Text.

Deep sequencing via cancer multigene panel testing revealed that the tumor contained point mutations, including *PTPRD* S1461*, *TP53* I255del, and *KDM6A* R1279*. In addition, copy number variations were identified, such as the amplification of chromosome 7, which includes *RAC1*, *ETV2*, *EGFR*, *MET*, and *BRAF*; amplification of the long arm of chromosome 20 (20q), which includes *BCL2L1* and *AURKA*; and amplification of *FGFR1* and *MYC* (Table 1).

**Table 1:** Pathogenic mutations and alterations of this case

Mutation								
Gene	chr	Start	End	Variant_type	HGVSc	HGVSp_AA	MAF	MUTATION_EFFECT
PTPRD	chr9	8389236	8389236	Nonsense_Mutation	c.4382C > A	p.S1461*	0.03	Likely loss-of-function
TP53	chr17	7577515	7577517	In_Frame_Del	c.764_766del	p.I255del	0.34	Likely loss-of-function
KDM6A	chrX	44950066	44950066	Nonsense_Mutation	c.3835C > T	p.R1279*	0.41	Likely loss-of-function

Alterations								
Gene	chr	Start	End	Seg. mean	seg.id	Number. targets	Type	Mutation effect
RAC1	chr7	6426875	6442153	0.40	22	8	Amplification	Gain-of-function
ETV1	chr7	13935460	14028710	0.40	22	15	Amplification	Gain-of-function
EGFR	chr7	55209944	55273331	0.40	22	30	Amplification	Gain-of-function
CDK6	chr7	92244419	92462652	0.39	24	6	Amplification	Gain-of-function
MET	chr7	116335761	116436216	0.39	24	25	Amplification	Gain-of-function
SMO	chr7	128843237	128852317	0.39	24	13	Amplification	Inconclusive
BRAF	chr7	140434379	140550017	0.39	24	20	Amplification	Gain-of-function
EZH2	chr7	148504682	148544404	0.39	24	21	Amplification	Gain-of-function
RHEB	chr7	151164229	151195282	0.39	24	7	Amplification	Likely Gain-of-function
FGFR1	chr8	38269901	38318680	0.35	25	22	Amplification	Gain-of-function
MYC	chr8	128748276	128753249	0.39	26	7	Amplification	Gain-of-function
BCL2L1	chr20	30253759	30310066	0.38	57	3	Amplification	Likely Gain-of-function
DNMT3B	chr20	31367881	31395734	0.38	57	23	Amplification	Likely Gain-of-function
SRC	chr20	36014456	36031788	0.38	57	11	Amplification	Likely Gain-of-function
TOP1	chr20	39658002	39751920	0.38	57	20	Amplification	Likely Gain-of-function
AURKA	chr20	54945120	54963303	0.38	57	10	Amplification	Gain-of-function

This study was approved by the Institutional Review Board of Hiroshima University Hospital (approval number: E-1869) and was conducted in accordance with the principles of the Declaration of Helsinki. Written informed consent was obtained from the patient.

## Discussion

We report a case of superficial ESCC with a circumferential, white-toned, elevated lesion formed by a thick keratinized layer. This lesion presented with pathological mutations in *TP53* and amplification of chromosomes 7 and 20q, *MYC*, and *FGFR1*, as identified by cancer genome analysis.

Ezoe et al. reported endoscopic findings of EEM summarized in four characteristic features: (1) a clear border with the surrounding area; (2) a slightly raised, white, flattened elevation; (3) a hairy or almost flat surface of the lesion; and (4) pale to indistinct staining with Lugol’s stain [5]. The diagnosis was relatively easy with an understanding of EEM. Elevated esophageal tumors include papillomas, keratoacanthomas, and glycogenic acanthoses. These tumors stain strongly with iodine, making them easy to differentiate from EEM, which does not stain with iodine. However, among elevated-type esophageal cancers, white-toned lesions with dyskeratosis make differentiation from EEM difficult. Differentiating EEM from verrucous carcinomas is particularly important. The characteristic endoscopic findings for esophageal verrucous carcinoma include an exophytic, cauliflower-like mass protruding into the esophageal lumen, with a surface often appearing white or grayish, a warty or verrucous texture, and a broad base with well-demarcated margins [6, 7]. Murawaki et al. reported that EEM presents with a translucent white color and a scaly, flat surface, whereas elevated esophageal cancer often appears opaque white with irregular surface undulations [8]. Yamanouchi et al. reported that the characteristic proliferative, exophytic, whitish, and wart- or cauliflower-like endoscopic findings of verrucous carcinoma may be useful for differentiating it from EEM [9].

This case presented with a clear border with the surrounding area and pale to indistinct staining with Lugol’s stain; however, it also had a tall, elevated lesion with roughness and irregularity. Therefore, it was difficult to differentiate it from verrucous carcinoma based on endoscopic findings alone. However, it showed features of EEM, such as a white, flat, elevated area and a shaggy surface, even in the more elevated ridges. The pathological findings of EEM are characterized by the presence of a thick keratinized layer on the superficial surface of the stratified squamous epithelium and a well-defined underlying granular layer [10]. In contrast, among squamous cell carcinomas, esophageal verrucous carcinoma has a highly differentiated papillary growth with a strong keratinizing tendency, characterized by the presence of atypical, albeit mild, pressure-drainage infiltrates [11].

Several potential reasons may explain the unique morphology observed in our case. The coexistence of cancer could be a potential cause; however, previous reports have not shown this specific morphology [9], suggesting that the likelihood of changes due to cancer coexistence is low. The factors contributing to the development of EEM are unknown; however, heavy smoking, excessive alcohol consumption, and gastroesophageal reflux disease have been identified as risk factors [11, 12]. In this case, chronic inflammation due to the accidental ingestion of caustic soda is considered a possible cause of EEM development. Furthermore, since the increase in EEM was observed after initiating EBD treatment for esophageal stenosis, it is possible that stimulation of the esophageal mucosa by EBD contributed to its proliferation. EBD is a procedure that involves deliberate injures to the esophageal mucosa for esophageal dilation, causing persistent mechanical irritation and chronic inflammation, which may promote EEM growth.

In addition to *TP53* and *KDM6A* mutations [13], which are commonly identified in ESCC, *PTPRD* mutations and *FGFR1* amplification, which are considered poor prognostic factors for ESCC, have also been identified [14, 15]. Thus, this case showed a considerably poor prognostic genomic landscape in terms of cancer genomics, which diverged from the pathological grade. Moreover, amplification of *MYC*, chromosome 7, and chromosome 20q was observed in this case. In our previous research, we reported that the primary mutations observed in superficial ESCC were TP53 and NOTCH1 mutations, CDKN2A deletions, and CCND1 amplifications. Amplifications of MYC chromosomes 7 and 20q were not observed in the genomic landscape of superficial ESCC [16]. MYC helps regulate the cell cycle and determines the proliferation rate [17]. Endo et al. reported that the genomic landscape of esophageal intraepithelial squamous cell neoplasia with epidermalization showed alterations commonly found in ESCC, such as *TP53* missense mutation, copy number gain of *PIK3CA*, and CN loss of *CDKN2A* [18]. Therefore, it is suggested that characteristic genomic alterations, such as amplification of *FGFR1* and *MYC* chromosomes 7 and 20q in this case, affected the unique morphological changes.

To the best of our knowledge, no reports have documented EEM cases presenting with high-elevated lesions similar to those observed in this case. In addition, this is the first report to conduct genomic analysis on an EEM case complicated by esophageal cancer. Although EBD is widely used for esophageal strictures with dysphagia, it is important to recognize the potential risk of EEM developing due to mechanical stimulation from EBD in esophageal strictures with EEM, as seen in this case. In addition, various genomic alterations can be involved in the growth of tumors into the esophageal lumen, suggesting the need for further investigation into the factors involved in the development and growth of EEM.

## Supplementary Information

Below is the link to the electronic supplementary material.

Supplementary file 1 (PDF 54 KB)

Supplementary file 2 (DOCX 16 KB)

## Data Availability

The data supporting this paper are available in the article and online supplementary material.
