# Supplementary material for: A case of esophageal squamous cell carcinoma with epidermization showing a unique morphology
Source: Clin J Gastroenterol. 2024 Oct 18;18(1):23–8. doi: 10.1007/s12328-024-02042-6 (PMC11785629; doi:10.1007/s12328-024-02042-6)
Supplement: Supplementary file 2 — (DOCX 16 KB) [file 12328_2024_2042_MOESM2_ESM.docx]

**Supplementary Text**

Sequencing reads were preprocessed using fastp v0.20 and mapped to hg19 using BWA-MEM v0.7.17.23. GATK best practice was used for variant calling. To reduce false positives, somatic mutations were defined as read depths >50 and variant allele frequencies >4%. Copy number analysis was performed using CNVkit v0.9.9 and PureCN v2.0.1. (1) Vcf2maf v1.6.21 (https://zenodo.org/record/1185418#.Y_W6cC_3IUs), oncokb-annotator v3.2.1 (https://github.com/oncokb/oncokb-annotator/releases), and InterVar v2.2.2 (2) were used for annotation. We defined alterations as mutations, amplifications, or deletions that are classified as oncogenic or likely oncogenic status in OncoKB (https://www.oncokb.org) or pathogenic or likely pathogenic status in ClinVar (https://www.ncbi.nlm.nih.gov/clinvar/). R package maftools v2.8.5 (https://bioconductor.org/packages/release/bioc/html/maftools.html) was used for plotting.

**References**

1. Riester M, Singh P, Brannon R, et al. PureCN: copy number calling and SNV classification using targeted short read sequencing. Source Code Biol Med. 2016;11:13.
2. Li Q, Wang K. InterVar: clinical interpretation of genetic variants by the 2015 ACMG-AMP guidelines. Am J Hum Genet. 2017;100:267–80
